# Analog and Digital Bipolar Resistive Switching in Co–Al-Layered Double Hydroxide Memristor

**DOI:** 10.3390/nano10112095

**Published:** 2020-10-22

**Authors:** Yanmei Sun, Li Li, Keying Shi

**Affiliations:** 1 Key Laboratory of Functional Inorganic Material Chemistry, Ministry of Education, School of Chemistry and Material Science, Heilongjiang University, Harbin 150080, China; sunyanmei@hlju.edu.cn; 2 Key Laboratory of Chemical Engineering Process & Technology for High-efficiency Conversion, School of Chemistry and Material Science, Heilongjiang University, Harbin 150080, China; 3 School of Electronic Engineering, Heilongjiang University, Harbin 150080, China

**Keywords:** hexazinone, Co–Al LDHs, resistive switching, nonvolatile memory

## Abstract

We demonstrate a nonvolatile memristor based on Co–Al-layered double hydroxide (Co–Al LDH). We also introduce a memristor that has a hexazinone-adsorbing Co–Al LDH composite active layer. Memristor characteristics could be modulated by adsorbing hexazinone with Co–Al LDHs in the active layer. While different, Co–Al LDH-based memory devices show gradual current changes, and the memory device with small molecules of adsorbed hexazinone undergo abrupt changes. Both devices demonstrate programmable memory peculiarities. In particular, both memristors show rewritable resistive switching with electrical bistability (>10^5^ s). This research manifests the promising potential of 2D nanocomposite materials for adsorbing electroactive small molecules and rectifying resistive switching properties for memristors, paving a way for design of promising 2D nanocomposite memristors for advanced device applications.

## 1. Introduction

For the past few years, more and more attention has been paid to memristors in the field of electronic devices [1,2,3,4,5,6,7,8,9,10,11,12]. They have been investigated extensively for commercialization on account of their quick response and data processing, and low energy consumption. Typical features of memristors are their two-terminal sandwich structure and resistive switching nonvolatile memories. According to the way the current increases, memristors can be roughly classified as digital or analog. The characteristic feature of a digital memristor is that resistive switching is abrupt, and it has been widely studied for its simple structure, easy three-dimensional stacking, and fast resistive switching speed [13,14,15,16,17,18]. For the last few years, analog memristors are typically characterized by gradual resistive switching, which has attracted more and more attention due to the application value of brain-like morphology calculations [19,20], programmable analog circuits [21], and the like. In particular, analog memristors present features with great promise in developing artificial synapses to implement brain-inspired neuromorphic computing. Broadly speaking, it is generally accepted by scholars that the mechanism of resistive switching is the formation and rupture of conductive filaments. A large number of filamentary memristors exhibit a sudden set or reset process, on behalf of their digital type. The opposite of that type is analog-type memristors [22]. At present, the mechanism of analog-type memristors is still unclear and controversial.

Since the memristor has great application potential in the electronics industry, much research is being devoted to commercialization of usable electronic components, which are largely concentrated in investigating different active layer materials [23,24,25]. According to previous reports, a variety of active layer materials have been studied, for example, binary oxides, organic polymers, and biomaterials [26,27,28]. In addition, in order to continuously improve the performance of memristor devices, researchers have used a variety of engineering methods to improve device performance, such as illumination effect [29,30], ultraviolet light exposure [31], multilevel data storage, sub-quantum filamentation, retention modeling by conductive bridge random access memory [31,32,33,34], defect control [35], atomic-level control with an optimized high temperature forming scheme [36], and so on. Therefore, development of new materials for study of memristors and control or adjustment of device performance is of great significance for development of nonvolatile memory. Recently, Co–Al-layered double hydroxide (Co–Al LDH) has attracted more and more attention for 2D composite materials on account of its big specific surface area and short carrier transport diffusion length [37,38]. The higher specific surface area makes it have a better adsorption capacity. Simultaneously, the hexazinone molecule contains an electroactive C=C–C=N group, which would bring about formation of an ingredient bearing two reducible centers; as a consequence, it has good reduction properties [39]. Given that triazines are electrochemically active, the hexazinone is a famous n-type semiconductor. Furthermore, it could be utilized as the adsorbed component in Co–Al LDH to regulate carrier migration. However, resistive switching biodevices using Co–Al LDH and their adsorption of small hexazinone molecules have not yet been reported.

Hence, in this work, the Co–Al LDH-based memristor and Co–Al LDH-adsorbed hexazinone-based memristor were fabricated to study resistive switching behavior and the effect of LDH adsorption of small molecules on their properties.

## 2. Materials and Methods

Co–Al LDH was synthesized as in our previous work [40,41]. 3-cyclohexyl-6-(dimethylamino)- l- methyl-1,3,5-Triazine-2,4-(1H,3H)-dione (hexazinone) (molecular weight: 252.31) was provided by Shanghai Fusheng Industrial Co., LTD. Co–Al LDH active layers were prepared by drop-casting technology on a glass substrate; Al vapor was deposited and dried at 50 °C for 8 h. The dry film was impregnated in 5 mg/mL hexazinone aqueous solution for 5 min and then dried at 50 °C overnight to form the Co–Al LDH-adsorbed hexazinone active layer. Whereafter, the electrode Al matrix was deposited by vacuum evaporation with the help of the mask (100 mm in diameter). Electrical properties of memristors were measured by a Keithley 4200 semiconductor parameter testing system. During measurement of the current–voltage (*I*–*V*) characteristics of the memristor, the bottom electrode was always grounded.

## 3. Results and Discussion

**Figure 1:**
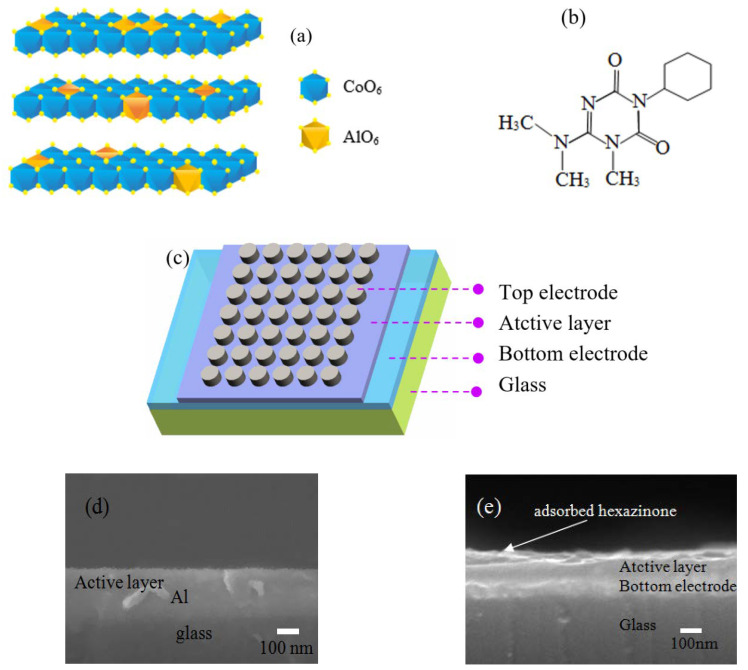
(**a**) Chemical structure diagram of Co–Al-layered double hydroxides (LDHs). (**b**) Chemical structure of hexazinone. (**c**) Schematic structure of the Co–Al LDHs-based memristor. (**d**) Cross section of the resistive switching layer for the Co–Al LDHs memristor. (**e**) Cross section of the resistive switching layer for the Co–Al LDH-adsorbed hexazinone memristor.

Memristors based on Co–Al LDHs or Co–Al LDH-adsorbed hexazinone as resistive switching active layers were fabricated on indium tin oxide (ITO)-coated glass substrates. Al/Co–Al LDH thin film/Al and Al/Co–Al LDH-adsorbed hexazinone thin film/Al structures were used to illustrate the memristors. Figure 1a depicts the chemical structure diagram of Co–Al LDHs, and Figure 1b depicts the chemical structure of hexazinone. Co–Al LDHs or Co–Al LDH-adsorbed hexazinone were used as the resistive switching layer in the devices. The schematic device structure of the Co–Al LDH-based memristor is shown in Figure 1c. The cross section of the resistive switching layer before deposition of top electrode Al was characterized by scanning electron microscopy. Figure 1d shows the cross section of the resistive switching layer for the Co–Al LDH memristor, and Figure 1e shows the cross section of the resistive switching layer for the Co–Al LDH-adsorbed hexazinone memristor.

**Figure 2:**
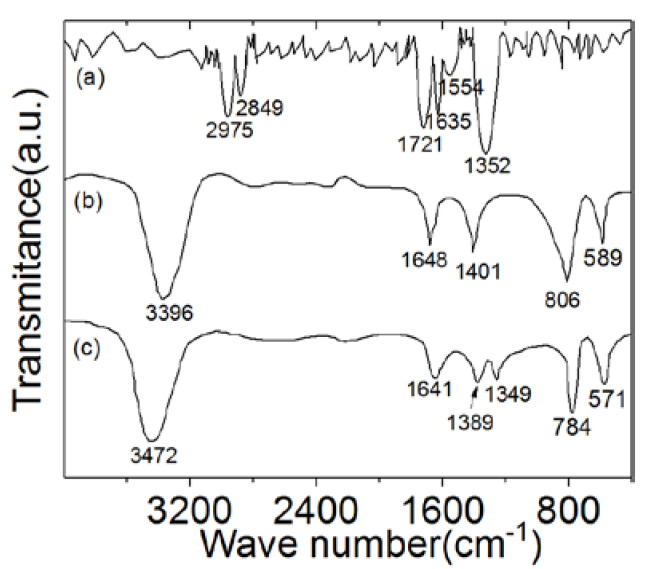
FTIR spectra of (**a**) hexazinone, (**b**) Co–Al LDHs, and (**c**) Co–Al LDHs after hexazinone adsorption.

FTIR spectra of hexazinone and Co–Al LDHs before and after hexazinone adsorption are shown in Figure 2. In the FTIR spectrum of hexazinone, as shown in Figure 2a, the absorption band at 2975 cm^−1^ corresponds to the stretching vibration of methyl C–H. Absorption bands centered at 2849 cm^−1^ were caused by the stretching vibration of methylene C–H. Two adjacent bands centered at 1721 and 1635 cm^−1^ were caused by the stretching vibration of 2-bit C=O and 1-bit C=O, respectively. On the other hand, absorption bands centered at 1554 cm^−1^ are attributed to the C=N stretching vibration. Absorption bands centered at 1352 cm^−1^ were caused by C–H deformation vibration of CH_3_O [39].

The broad strong absorption band centered at 3396 cm^−1^ originated from the stretching vibrations of surface and interlayer hydroxyl groups [42], as shown in Figure 2b. The weaker band at 1648 cm^−1^ came from the bending mode of water molecules. Bands centered at 589 cm^−1^ were derived from the stretching and bending vibration of metallic oxygen (M-O) or metallic hydroxyl (M-OH) [43]. The FTIR spectrum of Co–Al LDHs after adsorption of hexazinone is shown in Figure 2c. In hexazinone adsorbed on Co–Al LDHs, two bands at 2975 and 2849 cm^−1^ disappeared, and a new band of hexazinone appeared at 1349 cm^−1^, which indicates symmetric vibrations of hexazinone.

Resistive switching properties of Co–Al LDHs and the Co–Al LDH-adsorbed hexazinone memory device were accounted for in ambient conditions; for both devices, the tested *I–V* characteristics exhibited a typical nonvolatile memory property.

**Figure 3:**
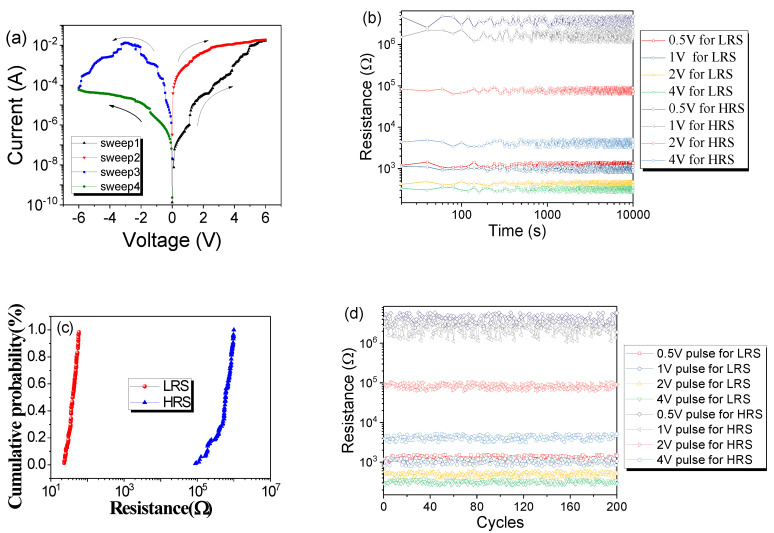
(**a**) *I–V* curves of the Co–Al LDHs memristor. (**b**) Data retention characteristics of the Co–Al LDHs memristor. (**c**) Cumulative probability for resistance in LRS and HRS under −2 V. (**d**) Endurance characteristics of the Co–Al LDH memristor.

Electrical characteristics of the Co–Al LDHs memristor were tested under the voltage sweep sequence of 0 V → +6 V, 0 V → +6 V, 0 V → −6 V, and then 0 V → −6 V. When sweeping positive external voltage from 0 to +6 V, the resistance converted gradually from high resistance state (HRS) to low resistance state (LRS). LRS could be maintained during the second positive voltage sweep until an opposite external voltage was applied LRS was gradually converted to HRS, and HRS was maintained during subsequent negative voltage scanning, as shown in Figure 3a. This indicates that the device has typical bipolar resistive switching behavior. For this memristor, the device maintains in LRS or HRS until an external voltage with opposite polarity is applied, which converts the resistance state to HRS or LRS. Furthermore, subsequent 10 consecutive voltage sweeps are shown in Supplementary Materials Figure S1. Repeatability of devices is an important prerequisite for their engineering applications. In the experiment, the *I–V* curves of 49 Co–Al LDHs memristor samples were tested, of which 30 samples had similar resistive properties and the remaining 19 did not. Sample-to-sample *I–V* characteristics for 30 Co–Al LDH memristors were shown in Figure S2. Additionally, the *I*–*V* plots of the remaining 19 are shown in Figure S3, as can be seen, and they only have a single resistive state and no resistive switching function. The calculation formula of power is p = *I* × *V*, where *I* represents the current and *V* represents the voltage. In this device, the current is 0.01 A at most, and the corresponding voltage is 6 V. Therefore, the maximum power consumption of these device is 0.06 watts.

Data retention characteristics of the Co–Al LDH memristor were carried out to evaluate the stability of the Co–Al LDHs memristor. Since resistive switching is gradual, we chose different voltages to motivate the device for HRS and LRS with excitation voltages of 0.5, 1, 2, and 4 V, respectively. This means that the current of the device is excited with different maximum voltages, and a reading voltage is used to read the resistance of the device. According to the resistive switching characteristics, the device can be converted to different resistance states when applying either a positive or negative applied voltage, so the excitation voltage can be either positive or negative. A reading constant voltage of 0.1 V was used to read the resistance of device, as shown in Figure 3b. It shows data retention characteristics of the Co–Al LDHs memristor. It can be seen that each resistance state of the Co–Al LDHs memristor is almost constant and can maintain a clear boundary with the adjacent resistance state. As a representative sample, data retention characteristics of the tenth Co–Al LDHs memristor are shown in Figure S4.

For the *I–V* curve of 30 Co–Al LDHs memristors in Figure S2, the cumulative probability for resistance in LRS and HRS under −2 V was collected, as shown in Figure 3c. The average resistance values required reflect its resistance level in LRS or HRS. Endurance characteristics of the Co–Al LDHs memristor were also carried out, as shown in Figure 3d. The pulse used had a pulse period and width of 2 and 1 ms, respectively. In order to reflect different resistance states of the Co–Al LDH memristor after excitation pulses of ±0.5, ±1, ±2, and ±4 V, respectively, were used, the 0.1 V reading voltage was used to read the resistance of the device. The Co–Al LDHs memristor was quite stable in every resistance state after 200 cycles. Endurance characteristics of the tenth Co–Al LDHs memristor are shown in Figure S5.

**Figure 4:**
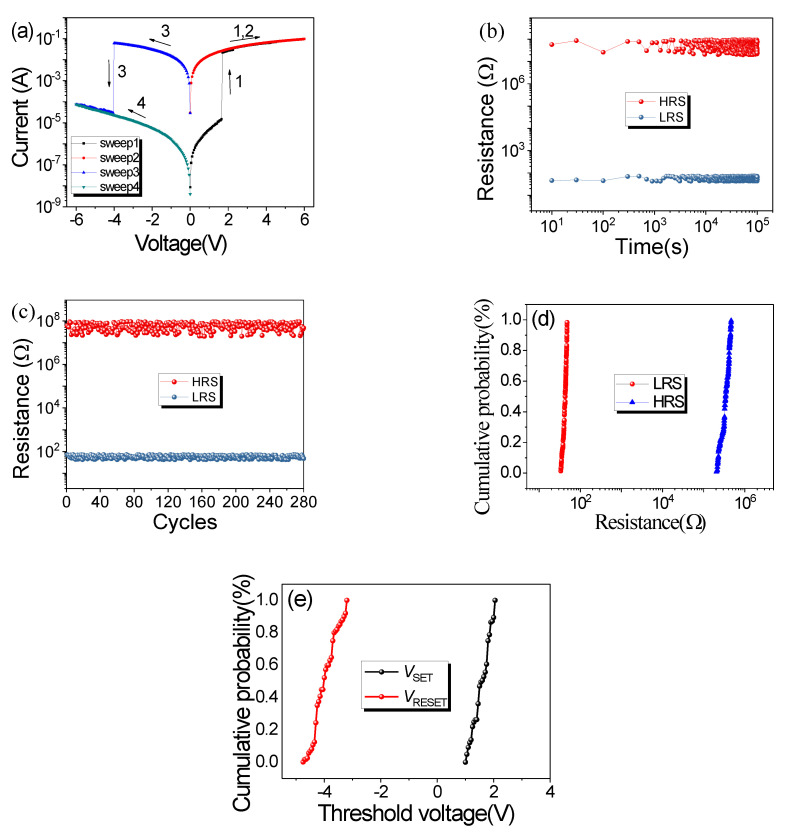
(**a**) *I–V* curves of the Co–Al LDH-adsorbed hexazinone memristor. (**b**) Data retention characteristics of the Co–Al LDH-adsorbed hexazinone memristor. (**c**) Endurance characteristics of the Co–Al LDH-adsorbed hexazinone memristor. (**d**) Cumulative probability for resistance in LRS and HRS. (**e**) Cumulative probability for threshold voltage in LRS and HRS.

Interestingly, in contrast to the Co–Al LDHs memristor, for the Co–Al LDHs after the hexazinone adsorption-based device, both the set and reset processes were abrupt (Figure 4a). In the Co–Al LDH-adsorbed hexazinone memristor, during the first positive voltage sweep from 0 V to the set voltage (*V*_set_) of 1.65 V the resistance state of the device was converted from its pristine HRS to LRS; this is the “set process” transition. The LRS was maintained during the subsequent positive voltage sweep. When a negative voltage was applied, which converted LRS to HRS at the *V*_reset_ of −4.05 V (“reset” process), the nonvolatile HRS and LRS were very stable, even when the power was turned off for 10 min or longer. The device could be converted over and over again between HRS and LRS by altering the applied voltage, and the subsequent 10 consecutive voltage sweeps are shown in Figure S6. In the experiment, the *I–V* curves of 56 Co–Al LDHs after the hexazinone adsorption-based device were tested, of which 39 samples had similar resistive properties and the remaining 17 did not. Sample-to-sample *I–V* characteristics for 39 Co–Al LDHs after hexazinone adsorption memristors are shown in Figure S7. The maximum power consumption of these devices is also 0.6 watts. In practice, the operating voltage can be reduced to reduce power consumption on the basis of ensuring the resistive switching characteristic.

Co–Al LDHs after the hexazinone adsorption-based device also exhibited stable data retention characteristics under the reading voltage of −0.1 V, as shown in Figure 4b, but with a high ON/OFF resistance ratio of 2.5 × 10^4^; after 10^5^ s, the device lost its data retention capability. Data retention characteristics of the tenth Co–Al LDHs after hexazinone adsorption memristors are shown in Figure S8. Figure 4c shows the endurance characteristics of the Co–Al LDH-adsorbed hexazinone memristor. For the endurance characteristics test, the set voltage pulse was +5 V, the reset voltage was set as −5 V, each pulse duration was 0.1 ms, and the set and rest pulse were followed by the reading pulse. The reading pulse had a pulse amplitude of 0.1 V. The resistance did not fluctuate significantly for the Co–Al LDH-adsorbed hexazinone memristor in both LRS and HRS during 280 read cycles, and after 280 cycles the resistive switching property was no longer repeated. Additionally, endurance characteristics of the tenth Co–Al LDHs after hexazinone adsorption memristor are shown in Figure S9. The 10^5^ s of data retention and 280 durable cycles of the device are far from meeting the requirements for practical application. Further improvement of data retention characteristics and endurance characteristics of the device is the next step that we need to focus on.

For the *I–V* curve of 39 Co–Al LDHs after hexazinone adsorption memristors in Figure S7, the cumulative probability for resistance in LRS and HRS were also collected for Co–Al LDHs after the hexazinone adsorption memristor, as shown in Figure 4d. The cumulative probability for threshold voltage of *V*_set_ and *V*_reset_ were also analyzed for Co–Al LDHs after the hexazinone adsorption memristor, as shown in Figure 4e. The average voltage required to accomplish set and reset operations was stable.

**Figure 5:**
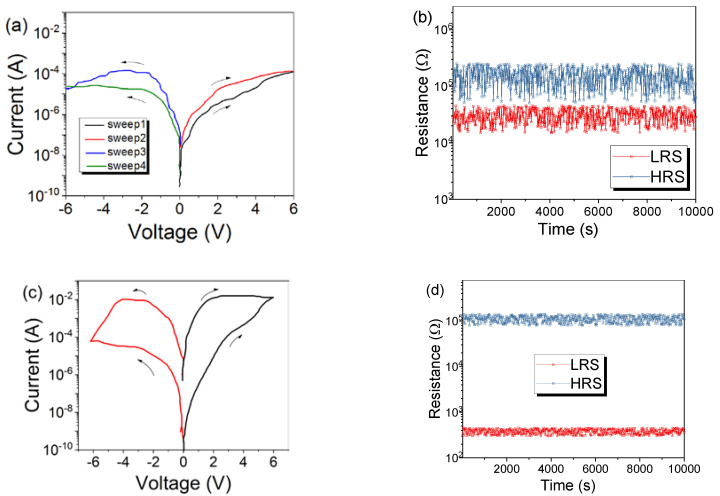
(**a**) *I–V* curves of the Co–Al LDHs memristor at 158 °C. (**b**) Data retention capability of Co–Al LDH memristor in HRS and LRS at −2 V constant voltage under 158 °C. (**c**) *I–V* curves of the Co–Al LDHs memristor at 85 °C. (**d**) Data retention capability of the Co–Al LDHs memristor in HRS and LRS at −2 V constant voltage under at 85 °C.

The influence of temperature on device performance has always been an important index in engineering applications. Considering that cycloqinone cannot withstand high temperatures, Co–Al LDHs memristor performance was tested at 158 °C, as shown in Figure 5. Figure 5a exhibits the *I–V* curves of the Co–Al LDHs memristor at 158 °C. It can be seen that the Co–Al LDHs memristor still exhibits gradual resistive switching at a high temperature. In contrast to room temperature conditions, the current in HRS and LRS significantly reduced, which may have been caused by the increase of resistance due to the intensified thermal motion of carrier molecules between the graded laminate structure of Co–Al LDHs at a high temperature. Figure 5b exhibits the data retention capability of the Co–Al LDHs memristor in HRS and LRS at −2 V constant voltage under 158 °C. With a minimum resistance ratio of about 2, the device almost loses its resistive switching capability. Such a low resistance ratio will greatly increase the misreading rate, which is far from meeting practical application requirements. Since the device does not have thermal stability at 158 °C, the device’s resistive switching and data retention characteristics are tested at 85 °C, as shown in Figure 5c,d. As can be seen, at this temperature the device has good temperature stability.

Data retention characteristics and endurance characteristics are important indicators to measure nonvolatile memory. In order to ensure security and reliability of data, the retention time of memory is generally required to be more than 10 years. As it is the next generation of nonvolatile memory, its endurance characteristics should exceed 10^6^. At present, only a few reported examples of resistive switching memory can meet this standard, and there is a big gap between our devices and this standard. In current researches on resistive switching memory, a variety of materials construct different resistive switching memory models, but the mechanism of resistive switching hidden behind different material systems is not clear enough. Resistive switching behaviors of different material systems also have their own characteristics, which indicate that resistive switching mechanisms of different systems are different.

At present, there are many models of a resistive switching mechanism proposed by the scientific community. The mechanism can be generally divided into two categories: interfacial effect and local effect. The resistive switching mechanism of interfacial effects includes an interfacial barrier regulator mode and a charge trap charge–discharge mode, while the resistive switching mechanism of local effects mainly includes metal conductive filaments, local valence state transition, electrothermochemical transition, and so forth. Therefore, the practical process of resistance random access memory needs a lot of further research.

**Figure 6:**
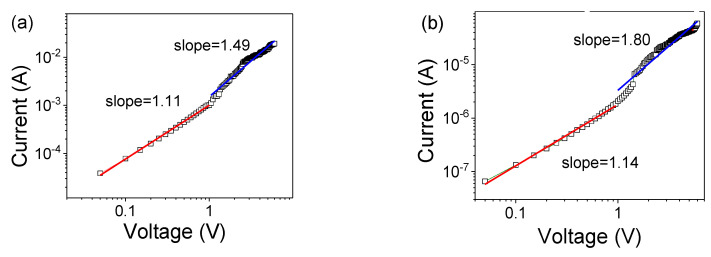

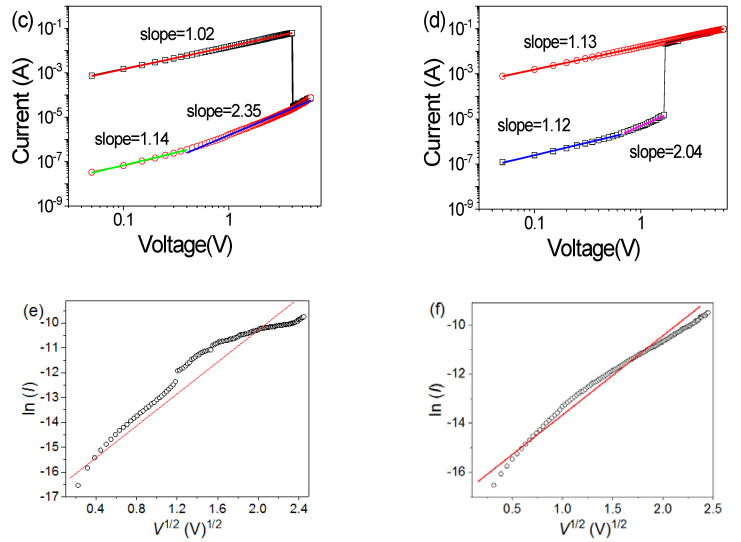
The log(*I*) µlog(*V*) plots with the Co–Al LDH memristor for space charge-limited conduction fitting for (**a**) HRS and (**b**) LRS. The log(*I*)µlog(*V*) plots for space charge-limited conduction fitting for the Co–Al LDH-adsorbed hexazinone memristor. (**c**) The negative voltage region. (**d**) The positive voltage region. The ln(*I*)µ*V*^1/2^ relationship fitting in HRS for (**e**) the Co–Al LDH memristor and (**f**) the Co–Al LDH-adsorbed hexazinone memristor.

To uncover the complex resistive switching mechanism of the device, the charge carrier transport of Co–Al LDHs and Co–Al LDH-adsorbed hexazinone were analyzed by model fitting of the *I−V* curves in the double logarithm coordinate as shown in Figure 6a–d. For the Co–Al LDH memristor, after data fitting we found that both HRS and LRS obey space charge-limited conduction [44], as shown in Figure 6a,b. For the adsorbed hexazinone memristor, the current and voltage relationship for the negative and positive voltage region was fitted under the log–log coordinate system, as shown in Figure 6c,d. We can see it in terms of the slopes of the HRS (the *I–V* relationship can be approximately described as *I–V* with slopes of 1.14 and 1.13 followed by *I–V*^2^ with slopes of 2.35 and 2.04) and LRS (the slopes were 1.02 and 1.13). The fitted current and voltage relationship can be ascribed to Ohm’s law for LRS and space charge-limited conduction for HRS. Additionally, the ln(*I*)µ*V*^1/2^ relationship fitting was performed for the Co–Al LDH memristor and Co–Al LDH-adsorbed hexazinone memristor, as shown in Figure 6e,f, indicating that the conduction behavior in the HRS for the Co–Al LDH memristor and Co–Al LDH-adsorbed hexazinone memristor can be ascribed to Schottky emission [45].

**Figure 7:**
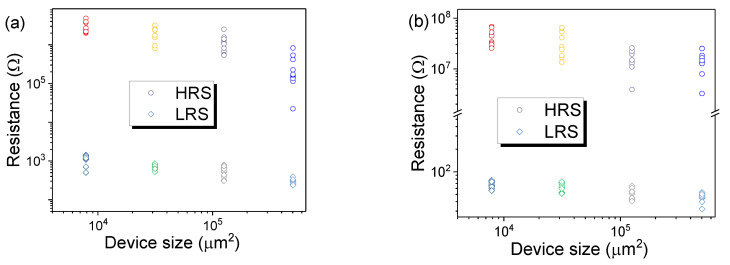
Device size dependence of the resistance for LRS and HRS for (**a**) the Co–Al LDH memristor and (**b**) the Co–Al LDH-adsorbed hexazinone memristor.

According to previous reports, the conductive filament theory is very popular [46,47]. According to this theory, resistance in LRS is independent of the size of the device. Therefore, device size dependence of the resistance in LRS and HRS for Co–Al LDHs and Co–Al LDH-adsorbed hexazinone-based memristors was performed, as shown in Figure 7. For each device size, resistance of 10 LRS and HRS tested from five devices were collected. The resistance was read at 0.2 V. According to the above experimental results, the conductive filament theory can be ruled out. Based on the above *I*–*V* characteristic fitting, the resistive switching mechanism is mostly attributed to Schottky emission.

**Figure 8:**
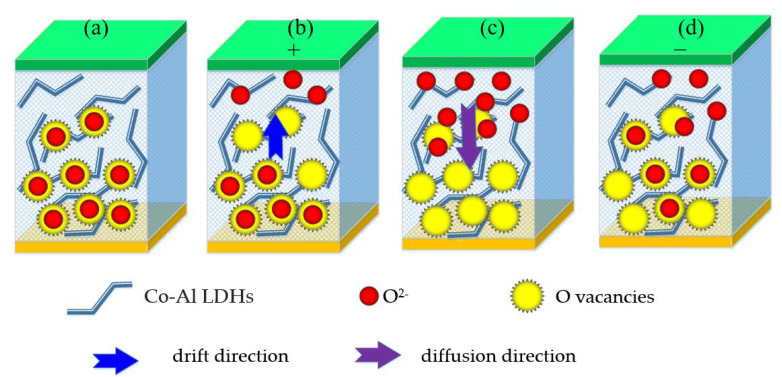
Schematic diagram of resistive switching of the Co–Al LDHs memristor. (**a**) Oxygen ion and oxygen vacancy when no voltage is applied to the device. (**b**) Oxygen ion drift when a positive voltage is applied on the Al electrode. (**c**) Diffusion of oxygen ions due to concentration gradients. (**d**) The trend of oxygen ion movement after applying negative voltage to Al electrode.

In consideration of low current density and gradual change in current, in combination with the above analysis for the mechanism of analog resistive switching, as previously reported, formation and migration of oxygen vacancies in LDHs are energetically expedient [48]. In addition, low electron affinity of oxygen (compared to its first ionization energy) may be conducive to formation of O^2−^ ions. Hence, we put forward here a simple model based on the drift-diffusion principle to explain the resistive switching process. Gradual change in the resistance state for the Co–Al LDHs memristor is due to drift and diffusion of oxygen ions and vacancies under applied voltage. A schematic diagram of resistive switching of the Co–Al LDHs memristor is shown in Figure 8. Figure 8a shows oxygen ion and oxygen vacancy when no voltage is applied to the device. For positive voltage applied to the Al top electrode, O^2−^ ions drift towards the Al top electrode, forming O vacancies among the Co–Al oxide octahedron, as shown in Figure 8b. These O^2−^ ions have a tendency to accumulate on the surface of Co–Al LDHs as a “sheet charge”. While O^2−^ ions may diffuse back into the dielectric because of the high concentration gradient, as shown in Figure 8c, the flux by reason of drift keeps most ions intact in the sheet charge. Hence, devices are gradually converted from HRS to LRS. In the negative voltage sweep, the drift effect has a tendency to gradually reduce, and the diffusion flux begins to dominate. This will lead to O^2−^ ions to diffuse back to passivate the O vacancies, as shown in Figure 8d, resulting in the drop of device conductivity, which converts devices from LRS to HRS.

**Figure 9:**
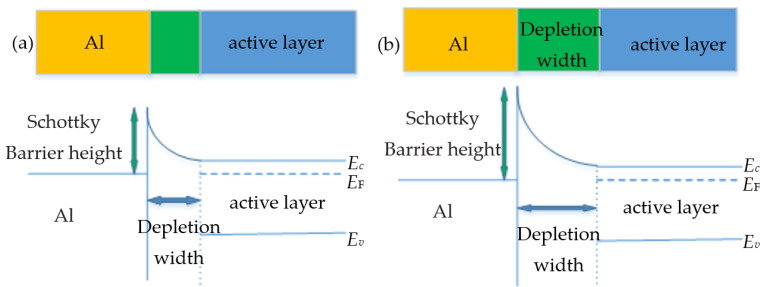
Schematic diagram of the change of Schottky barrier under the applied voltage to form a depletion layer: (**a**) under the positive voltage and (**b**) under the negative voltage.

The switching mechanism in Co–Al LDHs and its small-molecule adsorption material memristor is complicated, and more than one mechanism might run at the same time. Resistive switching is controlled by inherent characteristics of the LDH layer. After Co–Al LDH-adsorbed hexazinone, the sudden resistive switching behavior of the Co–Al LDH-adsorbed hexazinone memristor could be attributed to the strong reduction performance of hexazinone, cooperating with the drift/diffusion of oxygen ions and vacancies, along with the change of Schottky barrier under the applied voltage. Apart from drift/diffusion of oxygen ions and vacancies, when a positive voltage is applied to the top electrode (Al), the Schottky barrier between Al and Co–Al LDH-adsorbed n-type semiconductors hexazinone will be reversed to form a depletion layer, which is formed at the interface between the metal Al and the active layer. The schematic diagram of the change of Schottky barrier under the applied voltage to form a depletion layer is shown in Figure 9. In addition, a large number of majority carriers (electrons) produced by the ionization of n-type semiconductors of the small molecule hexazinone will migrate toward the top electrode under a positive electrical field. The electrons are attracted by a positive voltage through the depletion layer. As a consequence, the depletion layer was narrowed, leading to reduction in the height of the Schottky barrier. By contrast, when the negative voltage is applied to the top electrode, electrons migrate back to the Co–Al LDH bulk near the interface, which recombines with oxygen vacancies, leading to increase in the Schottky barrier height. After Co–Al LDH-adsorbed hexazinone, more charge carriers migrate toward the electrode accompanied by broadening of the depletion layer. Therefore, conductivity of the device is altered more significantly, which makes the resistive switching process abrupt. Azomethine moieties in hexazinone as donors will deplete further [49]. At present, the academic community has not reached a consensus on the mechanism of resistive switching. Researchers have proposed a variety of mechanisms to explain the resistive switching behavior, and some microscopic confirmatory experiments are lacking in this work to clarify the resistive switching mechanism.

Performance comparison of resistive switching memory between the Co–Al LDH-adsorbed hexazinone-based devices (this work) and other 2D material-based memory devices reported by several research groups was performed, as shown in Table 1. In comparison with the materials of BiOI, PCBM–MoS_2_ nanocomposites, 2D/3D heterostructure-based CH_3_NH_3_PbI_3-x_Cl_x_, and hexagonal boron nitride, our Co–Al LDH-adsorbed hexazinone-based device has a higher ON/OFF resistance ratio of 2.5 × 10^4^. Furthermore, our device has the highest data retention characteristics (10^5^ s) compared to other materials in Table 1.

## 4. Conclusions

In conclusion, on the basis of drop coating and impregnation methods to fabricate Co–Al LDHs and Co–Al LDH-adsorbed hexazinone-based memristors, the memory behavior of memristors rectified by adsorbed hexazinone was observed. Impressively, Co–Al LDH-based memory devices exhibit gradual changes in current, while the memory device based on adsorbed hexazinone small molecules shows abrupt changes in current. Both memristors show rewritable resistive switching with superior electrical bistability (>10^5^ s). The strategies implemented here, including the 2D nanocomposite material that adsorbed electroactive small molecules for adjusting the resistive switching property of hybrid nanomaterials, could principally be extended to other systems of adsorbing electroactive small molecules and offer unique solutions for adjusting the resistive switching behavior of 2D hybrid nanomaterials.

## Figures and Tables

**Table 1:** Performance comparison of resistive switching memory based on 2D materials.

Materials	Resistive Switching	Data Retention Characteristics	Endurance Characteristics (Cycles)	ON/OFF Resistance Ratio	Ref.
BiOI	Rewritable	1.4 × 10^4^ s	100	~10	[50]
PCBM–MoS_2_ nanocomposites	Rewritable/WORM	1.0 × 10^4^ s	95	~3 × 100 (rewritable)	[10]
Layered K-Birnessite nanosheets	Nonvolatile memory switching/volatile threshold switching	3.0 × 10^4^ s	800	~2 × 10^5^ (memory switching)	[51]
2D/3D heterostructure-based CH_3_NH_3_PbI_3-x_Cl_x_	Rewritable	1.2 × 10^4^ s	300	10^3^	[52]
Hexagonal boron nitride	Bipolar and threshold resistive switching	10–100 s	200	10^2^	[53]
Co–Al LDH-adsorbed hexazinone	Rewritable	10^5^ s	280	2.5 × 10^4^	This work

## Supplementary Materials

The following are available online at https://www.mdpi.com/2079-4991/10/11/2095/s1, Figure S1: The subsequent 10 consecutive voltage sweeps for CoAl-LDHs memristor, Figure S2: Sample-to-sample *I-V* characteristic for 30 CoAl-LDHs memristors, Figure S3: Representative *I-V* plots of the remaining 19 devices which show a single resistive state. (a) *I-V* characteristic of the first device. (b) *I-V* characteristic of the seventh device. (c) *I-V* characteristic of the sixteenth device, Figure S4: Data retention characteristics of the tenth Co-Al LDHs memristor, Figure S5: Endurance characteristics of the tenth Co-Al LDHs memristor, Figure S6: The subsequent 10 consecutive voltage sweeps for CoAl-LDHs after hexazinone adsorption memristor, Figure S7: Sample-to-sample I-V characteristic for 39 CoAl-LDHs after hexazinone adsorption memristors, Figure S8: Data retention characteristics of the tenth CoAl-LDHs after hexazinone adsorption memristors, Figure S9: Endurance characteristics of the tenth CoAl-LDHs after hexazinone adsorption memristor.
