# Supplementary material for: Analog and Digital Bipolar Resistive Switching in Co–Al-Layered Double Hydroxide Memristor
Source: Nanomaterials (Basel). 2020 Oct 22;10(11):2095. doi: 10.3390/nano10112095 (PMC7690433; doi:10.3390/nano10112095)
Supplement: Supplementary file 1 [file nanomaterials-10-02095-s001.pdf]

Supporting Information

Analog and digital bipolar resistive switching in Co-Al layered double hydroxide memristor

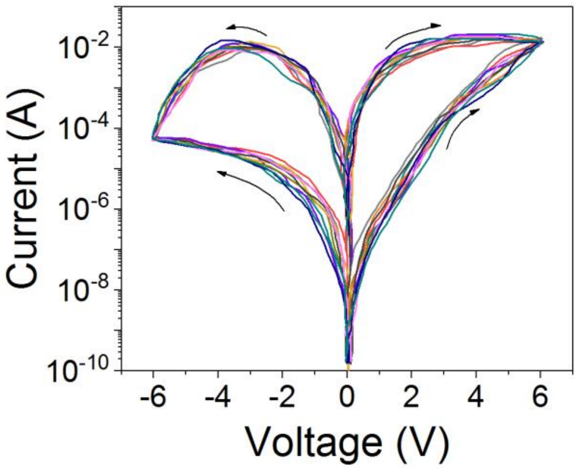

Figure S1. The subsequent 10 consecutive voltage sweeps for CoAl-LDHs memristor.

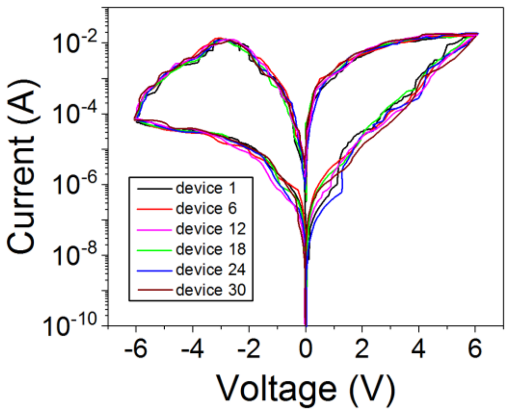

Figure S2. Sample-to-sample *I-V* characteristic for 30 CoAl-LDHs memristors.

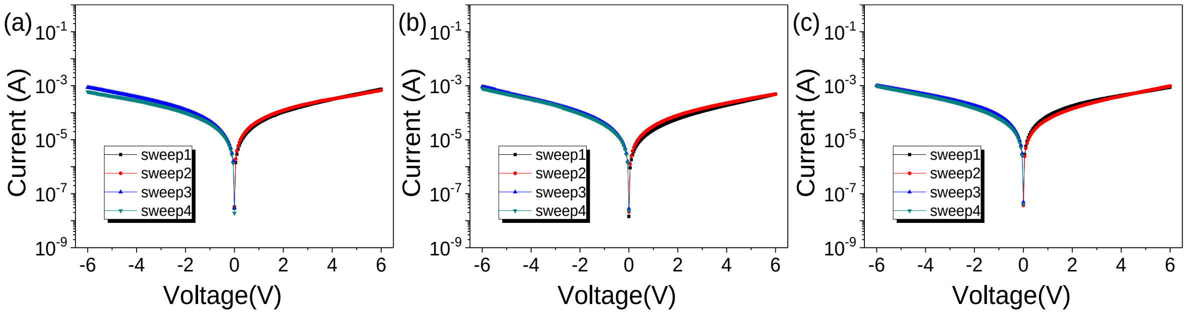

Figure S3. Representative I-V plots of the remaining 19 devices which show a single resistive state. (a) I-V characteristic of the first device. (b) I-V characteristic of the seventh device. (c) I-V characteristic of the sixteenth device.

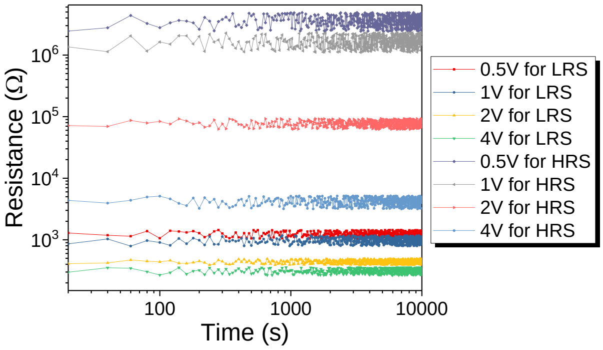

Figure S4. Data retention characteristics of the tenth Co-Al LDHs memristor.

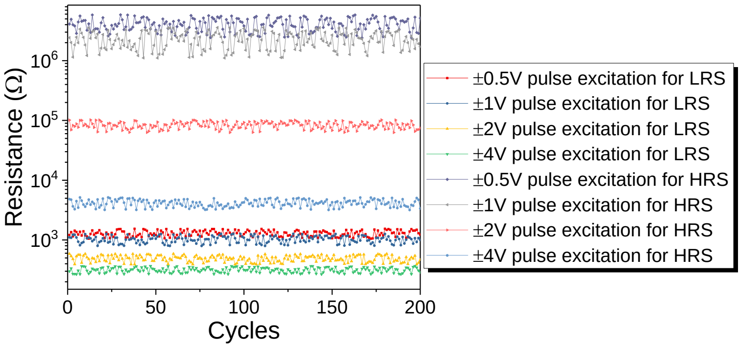

Figure S5. Endurance characteristics of the tenth Co-Al LDHs memristor.

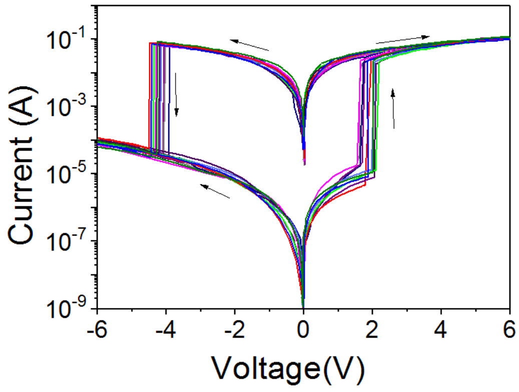

Figure S6. The subsequent 10 consecutive voltage sweeps for CoAl-LDHs after hexazinone adsorption memristor.

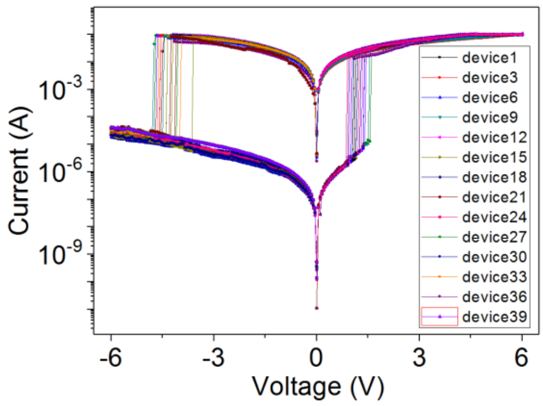

Figure S7. Sample-to-sample *I-V* characteristic for 39 CoAl-LDHs after hexazinone adsorption memristors.

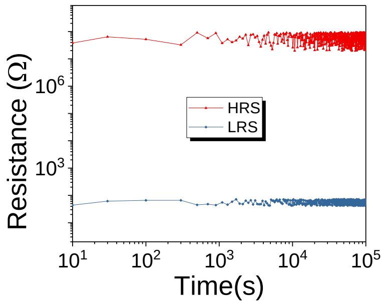

Figure S8. Data retention characteristics of the tenth CoAl-LDHs after hexazinone adsorption memristors.

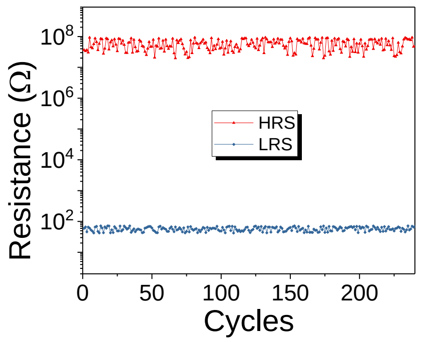

Figure S9. Endurance characteristics of the tenth CoAl-LDHs after hexazinone adsorption memristor.
